# Cross-cultural adaptation of VISA-P score for patellar tendinopathy in Turkish population

**DOI:** 10.1186/s40064-016-3100-x

**Published:** 2016-08-30

**Authors:** Mehmet Mesut Çelebi, Serdal Kenan Köse, Zehra Akkaya, Ali Murat Zergeroglu

**Affiliations:** 1 Sports Medicine Department, School of Medicine, Ankara University, 06590 Cebeci, Ankara, Turkey; 2 Biostatistics Department, School of Medicine, Ankara University, 06590 Cebeci, Ankara, Turkey; 3 Radiology Department, School of Medicine, Ankara University, 06590 Cebeci, Ankara, Turkey

**Keywords:** VISA-P-Tr, Patellar tendinopathy, Reliability, Validity

## Abstract

**Introduction and purpose:**

VISA-P questionnaire assesses to severity of symptoms and treatment effects in athletes with patellar tendinopathy. The purpose of this study was to translated VISA-P questionnaire into Turkish language and to determine its validity and reliability.

**Methods:**

The English version of VISA-P questionnaire was translated into Turkish according to the internationally recommended guidelines. Test–retest reliability was determined on 89 participants with time interval 24 h. To determine validity of Turkish VISA-P, 31 (17 male, 14 female) healthy students, 34 (20 male, 14 female) patients with patellar tendinopathy (diagnosed by physical examination and ultrasonography) and 24 (16 male, 8 female) volleyball players (at risk populations) were completed VISA-P-Tr. Internal consistency was determined with Cronbach’s alpha. Intraclass correlation coefficients (ICCs) were calculated to analyse test–retest reliability. To assessment of discrimination, VISA-P-Tr scores compared all groups using the Mann–Whitney-U test.

**Results:**

The VISA-P-Tr questionnaire showed good test–retest reliability (The Cronbach’s alpha was 0.79 and 0.78 respectively and ICC was 0.96). The VISA-P-Tr score (mean ± SD) were 93.7 ± 8.9 and 94.0 ± 8.1 for healthy students, 81.1 ± 13.7 and 80.7 ± 13.4 for volleyball players, 58.8 ± 12.1 and 58.5 ± 11.0 for athletes with patellar tendinopathy.

**Conclusion:**

The translated Turkish version of VISA-P has good internal consistency and good reliability and validity. Therefore VISA-P-Tr is useful to evaluate symptoms and follow the treatment effect in athletes with patellar tendinopathy.

## Background

Patellar tendinopathy (also known as jumpers knee) is common injury in athletes especially running and jumping athletes, such as volleyball players, basketball players and track and field athletes (Khan 1998; Kongsgaard et al. 2009; Van Der Worp et al. 2011). It is believed that repetitive and continuous stress causes that injury. Patellar tendinopathy prevalence is especially high among these athletes (Lian et al. 2005). Physical examination and ultrasound can be used for diagnosis and evaluation of to severity of symptoms but good correlation may not always be obtained. Valid and reliable tools are required for clinical use to evaluate for severity of symptoms, functional tests and treatment effect (Khan 1998). The Victorian Institute of Sports Assessment from Austria was developed a questionnaire for assess severity of patellar tendinopathy in athletes in 1998 and it is called VISA-P (Visentini et al. 1998). VISA-P also can be used to assess treatment effects. Since the publication of this questionnaire, it was adapted by several populations such as German, Spanish, Korean, Greek, Italian, Dutch, Brazil, Swedish and French populations (Frohm et al. 2004; Hernandez-Sanchez et al. 2011; Korakakis et al. 2014; Lohrer and Nauck 2011; Maffulli et al. 2008; Maher et al. 2007; Park et al. 2013; Wageck et al. 2013; Zwerver et al. 2009; Kaux et al. 2016). It is widely accepted method for assessment of patellar tendinopathy. The purpose of this study was to translate VISA-P questionnaire into Turkish language and to determine the validity and reliability of the Turkish version.

## Methods

We informed developers of original VISA-P questionnaire and asked their consent (Personal communication with J Cook). VISA-P questionnaire is consist of eight questions. First 6 questions are to evaluate severity of symptoms during sportive activities, and last two questions are ask information interaction and participations about sports (Visentini et al. 1998). First 7 question of VISA-P questionnaire has maximum score of 10 points, and last question has 30 points. Theoretically VISA-P questionnaire has 100 maximum points and 0 minimum point.

### Cross cultural adaptations

The English version of VISA-P questionnaire was translated into Turkish according to the internationally recommended guidelines (Beaton et al. 2000; Sperber 2004). Cross-cultural adaptation was performed according to international recommendation and it’s consisting of 6 following steps (Translation, synthesis, back translations, expert committee reviewing, pretesting and validations) (Beaton et al. 2000). First and second steps were performed by two bilingual individual whose native language was Turkish. Both of them were medical doctors whose clinical expertise was in musculoskeletal disorders. Back translations were performed by two people who were bilingual but not familiar with VISA-P questionnaire. One of them is doctoral student and one of them is radiologist both of whom are not familiar VISA-P. An expert team with members are consist of all translators and research team (2 sport medicine physicians, orthopedist, radiologist and statistical analyzer) After team members reached their final verdict, final version of the questionnaire was performed for pretesting. We consulted 10 participants involved in different sports (soccer, volleyball and basketball players). After re-evaluated, expert committee added 2 schematic figures question 3 and 4. Final version of VISA-P-Tr was indicated. Validation study details and results are provided below (“Appendix”).

#### Validity

Face validity refers relevant information of test included in the questionnaire. In our study, face validity was tested by authors and participant.

Content validity refers the questionnaire has appropriate indicators of construct. Expert committee has decided content correlated with clinical findings according to literature (Frohm et al. 2004; Hernandez-Sanchez et al. 2011; Korakakis et al. 2014; Lohrer and Nauck 2011; Maffulli et al. 2008; Maher et al. 2007; Park et al. 2013; Wageck et al. 2013; Zwerver et al. 2009; Kaux et al. 2016).

For assessing known group validity, VISA-P-Tr scores were compared healthy group and risk and patellar tendinopathy groups using Mann–Whitney U test with Bonferroni correction for avoiding possible statistical errors.

Convergent validity is a sub-part of concurrent validity and it is usually demonstrated by correlation two or more measurement. For assessing convergent validity we analysed correlation between clinical finding (pain, participation to sport) with VISA-P score. We evaluate the pain with visual analog scale (VAS) score.

Responsiveness refers to measure of the changes individual values over of time period. To assess responsiveness we used paired t test.

We measured the ceiling and floor effect of VISA-P-Tr questionnaire. If 15 % of the participants reached theoretically maximum or minimum of total scores we considered to have a ceiling or floor effect. For each question of on the questionnaire if participant reached 75 % maximum score we considered ceiling effect, and reached 75 % minimum score we considered floor effect.

Factorial validity was studied of questionnaire with using Confirmative Factor Analysis.

To assessment of discrimination, VISA-P-Tr scores compared all groups using the Mann–Whitney-U test.

For internal consistency was calculated for at risk and tendinopathy groups.

#### Reliability

Test–retest reliability was determined on 89 participants with a time interval of 24 h. 31 (17 male, 14 female) healthy students, 34 (20 male, 14 female) patients with patellar tendinopathy (diagnosed by with physical examination and ultrasonography) and 24 (16 male, 8 female) volleyball players (at risk groups) were completed VISA-P-Tr. Internal consistency was determined with Cronbach’s alpha. Intraclass correlation coefficients (ICCs) were calculated to analyse test–retest reliability.

#### Subjects

31 (17 male, 14 female) healthy students, 34 (20 male, 14 female) patients with patellar tendinopathy (diagnosis with physical examination and ultrasonography) and 24 (16 male, 8 female) volleyball players (at risk populations) were completed VISA-P-Tr (Table 1).

**Table 1:** Descriptive characteristics of subjects

	Healthy	At risk	PT
Age (year)	24.3 ± 3.6	28.1 ± 5.4	21.8 ± 5.8
Gender (m:f)	17:14	16:8	20:14
Height (cm)	172.1 ± 10.4	190.2.1 ± 9.1	186.1 ± 9.3
Weight (kg)	71.6 ± 17.2	83.3 ± 10.8	75.1 ± 11.7
BMI (kg/m^2^)	23.9 ± 3.9	22.9 ± 1.6	21.5 ± 3.3

We decided to the number of subjects according to the method by Walter et al. (1998).

Design: To assess test–retest reliability all participant filled out the VISA-P-Tr twice (before and 24 h after physical examination). All participant were evaluated through a physical examination and ultrasonography for diagnosis of patellar tendinopathy.

After diagnosis, all patient with patellar tendinopathy were treated at our department. Our treatment protocol focus on strengthening of the muscles around the knee in subjects with jumper’s knee, and measuring changes in strength, pain, and function after an eight intervention.

### Statistical analyses

All data are represented mean ± SD. Statistical significance was set p < 0.05.

#### Validity

To assessment of discrimination, VISA-P-Tr scores compared all groups using the Mann–Whitney-U test. SPSS ver 11.5 (SPSS Inc., Chicago, IL, USA) was used for statistical analysis. Convergent validity was studied of questionnaire with using Pearson correlation test. Factorial validity was studied of questionnaire with using Confirmative Factor Analysis. Goodness of fit indexes was also given.

#### Reliability

Internal consistency was determined with Cronbach’s alpha (Cronbach and Meehl 1955). Intraclass correlation coefficients (ICCs) were calculated to analyse test–retest reliability.

Ankara University Human Participant Research Ethics Committee approved all procedures used in this investigation.

## Results

During translation period, minor problems were solved by the expert committee.

VISA-P-Tr scores of all subjects summarized in Table 2.

**Table 2:** VISA-P-Tr Scores

	Healthy test (n = 31)	Healthy re-test (n = 31)	At risk test (n = 24)	At risk re-test (n = 24)	PT test (n = 34)	PT re-test (n = 34)
Q1	9.4 ± 1.5	9.5 ± 1.2	9.1 ± 1.9	9.0 ± 1.9	6.9 ± 2.7	6.7 ± 2.7
Q2	9.8 ± 0.6	9.8 ± 0.6	8.5 ± 2.4	8.3 ± 2.4	6.0 ± 2.6	6.0 ± 2.8
Q3	9.6 ± 1.1	9.6 ± 1.1	8.5 ± 2.2	8.8 ± 2.1	6.4 ± 2.4	5.8 ± 2.8
Q4	9.5 ± 1.1	9.4 ± 1.2	7.6 ± 2.7	7.2 ± 2.9	4.6 ± 2.2	4.7 ± 2.2
Q5	9.5 ± 1.1	9.5 ± 0.9	7.4 ± 2.5	7.2 ± 2.8	4.8 ± 2.9	5.0 ± 2.5
Q6	9.2 ± 1.1	9.5 ± 0.9	7.6 ± 2.8	7.3 ± 3.2	4.7 ± 2.5	4.7 ± 2.5
Q7	9.8 ± 0.8	9.8 ± 0.8	9.0 ± 1.9	8.8 ± 2.0	8.4 ± 2.2	8.3 ± 2.5
Q8	26.7 ± 7.0	26.1 ± 7.6	23.8 ± 5.8	23.8 ± 5.8	18.0 ± 4.1	17.7 ± 5.3
Total	93.7 ± 8.9	94.0 ± 8.1^a^	81.1 ± 13.7	80.7 ± 13.4^a^	58.8 ± 12.1	58.5 ± 11.0*, **

*PT* patellar tendinopathy, *Q* question

* PT group significantly lower than healthy ** and at risk group (p < 0.05) with using Mann–Whitney U

^a^Intraclass correlation coefficients (ICC) between test re-test = 0.96

Known group validity was demonstrated by significantly lower scores for tendinopathy group compared healthy and at risk group (p < 0.05) (Table 2).

**Fig. 1:**
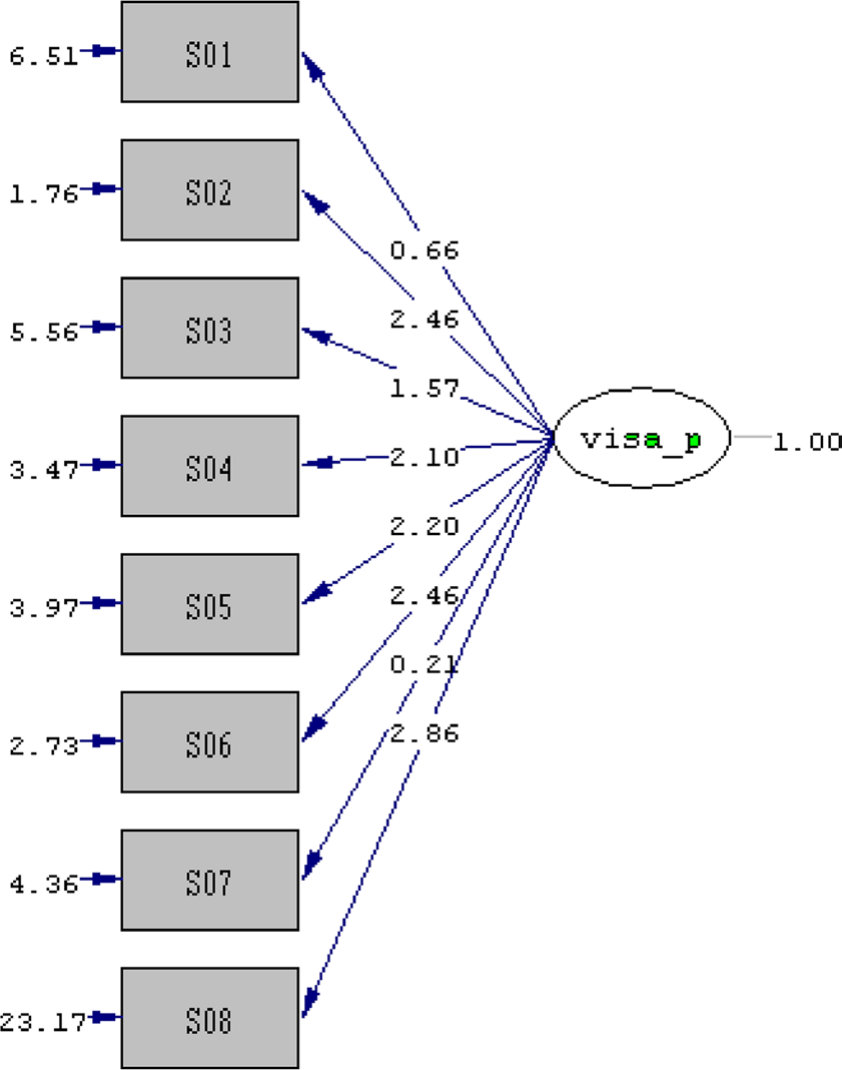
Result of Confirmative Factor Analysis. Goodness of fit index (GFI) = 0.88

Goodness of fit index was used for factorial validity. Goodness of fit index should be between 0-1 for significance. Our result was 0.88 which was very good (Fig. 1).

Only 11.2 % of participants reached maximum scores and nobody had minimum scores. For each items on the questionnaire no item received more than 75 % of minimum or maximum participants. Ceiling and floor effects were not found in this study.

Convergent validity were between VAS and VISA-P-Tr score: r = 0.473 p ≤ 0.0001, and participation of sports and VISA-P-Tr score: r = 0.419 p ≤ 0.0001.

During assessment of responsiveness no significant changes found all groups first and second VISA-P-Tr.

For internal consistency, Cronbach’s alpha coefficients were 0.79 and 0.78 respectively. ICC was 0.96.

## Discussion

It was no significant problem reported during translation period. Minor problems were solved by the expert committee. After first version of VISA-P-Tr, we consulted 10 participants involved in different sports (soccer, volleyball and basketball players). They did not understand correctly question 3 and 4. They misunderstood question 3 and 4. Some of participant did not know what lunge mean is. They suggested to add schematic figures question 3 and 4. After adding 2 figures, there were not any problem during translation process and adaptation to Turkish.

Our study revealed that the cross-cultural adaptation and the validation of VISA-P-Tr can be conducted successfully according to the guidelines suggested by Beaton et al. (2000).

Our study, test–retest reliabilities of questionnaire (ICCs) were 0.96 for 24 h intervals.

The VISA-P-Tr results were consistent with other studies (Table 3) (Frohm et al. 2004; Hernandez-Sanchez et al. 2011; Korakakis et al. 2014; Lohrer and Nauck 2011; Park et al. 2013; Zwerver et al. 2009).

**Table 3:** VISA-P–Tr scores compared with original and other adapted version scores

	Healthy	At-risk	Tendinopathy
Current study	93.7 ± 8.9 n = 29	81.1 ± 3.7 n = 24	58.8 ± 12.1 n = 34
Dutch Group (Maher et al. 2007)	95.3.6 ± 8.8 n = 18	88.6 ± 11.1 n = 15	58.2 ± 18.9 n = 14
English Group (Visentini et al. 1998)	95.0 ± 8.0 n = 18	93.0 ± 11.0 n = 100	55.0 ± 12.0 n = 14
German Group (Lohrer and Nauck 2011)	94.8 ± 6.3 n = 57	93.0 ± 7.0 n = 15	62.3 ± 13.0 n = 23
Greek Group (Korakakis et al. 2014)	95.0 ± 6.7 n = 61	97.9 ± 3.7 n = 64	53.3 ± 8.1 n = 32
Korean Group (Park et al. 2013)	92.6 ± 8.6 n = 5	No data	67.6 ± 15.7 n = 23
Spanish Group (Hernandez-Sanchez et al. 2011)	95.4 ± 2.5 n = 40	90.0 ± 9.7 n = 40	54.8.2 ± 13.3 n = 40
Swedish Group (Frohm et al. 2004)	83.1 ± 12.6 n = 17	79.0 ± 24.2 n = 17	47.7 ± 20.2 n = 17
French Group (Kaux et al. 2016)	99 ± 2 n = 22	86 ± 14 n = 42	53 ± 17 n = 28

n = sample size

Correlation between symptoms and VISA-P-Tr scale would be evidence of convergent validity.

If we compared VISA-P-Tr scores other version of VISA-P-scores; healthy and tendinopathy groups scores were consistent with other studies except Swedish Group. Swedish Group scores are lower than all groups. For the risk group, our scores were consistent with the results of Swedish group and French group (Frohm et al. 2004; Kaux et al. 2016). The lower VISA-P-Tr scores at risk groups possibly reflects their sports ages. Swedish group has also lower tendinopathy scores than other studies.

This was one of limitations of this study we did not evaluate VISA-P-Tr long term effect. Our interval between test re-test was 24 h. Korean groups used 2 h (short term) and 1 week (long term) (Park et al. 2013). Italian group used retest after 30 min (Maffulli et al. 2008). German groups used for test re-test reliability 24 h. We believed that 30 min and 2 h too short for excluded memory effect. But, if you assess treatment effect you have to use long term re-test interval. Responsiveness can be use to evaluate treatment effect. There was no change found assessing responsiveness at our study. Only Spanish group was evaluated responsiveness their study. They evaluated PT group after 15–17 days interval and found that VISA-P-Sp can be used for treatment effect. In the future we are planning to evaluate responsiveness to following treatment effect of PT.

Our study internal consistency score was 0.79, which was consistent with other studies.

## Conclusion

We concluded that the translated Turkish version of VISA-P has good internal consistency and good reliability and validity. Therefore VISA-P-Tr is useful to evaluate symptoms and follow the treatment effect in athletes with patellar tendinopathy.
